# PARP inhibitors: enhancing efficacy through rational combinations

**DOI:** 10.1038/s41416-023-02326-7

**Published:** 2023-07-10

**Authors:** Deepak Bhamidipati, Jaime I. Haro-Silerio, Timothy A. Yap, Natalie Ngoi

**Affiliations:** 1 Department of Cancer Medicine Fellowship Program, The University of Texas MD Anderson Cancer Center, Houston, TX USA; 2 Department of Internal Medicine, Baylor College of Medicine, Houston, TX USA; 3 Department of Investigational Cancer Therapeutics (Phase I Program), The University of Texas MD Anderson Cancer Center, Houston, TX USA; 4 The Institute for Applied Cancer Science, University of Texas MD Anderson Cancer Center, Houston, TX USA; 5 Department of Haematology-Oncology, National University Cancer Institute, Singapore, Singapore

**Keywords:** Targeted therapies, Drug development

## Abstract

Poly (ADP-ribose) polymerase inhibitors (PARPi) have significantly changed the treatment landscape for tumours harbouring defects in genes involved in homologous repair (HR) such as *BRCA1* and *BRCA2*. Despite initial responsiveness to PARPi, tumours eventually develop resistance through a variety of mechanisms. Rational combination strategies involving PARPi have been explored and are in various stages of clinical development. PARPi combinations have the potential to enhance efficacy through synergistic activity, and also potentially sensitise innately PARPi-resistant tumours to PARPi. Initial combinations involving PARPi with chemotherapy were hindered by significant overlapping haematologic toxicity, but newer combinations with fewer toxicities and more targeted approaches are undergoing evaluation. In this review, we discuss the mechanisms of PARPi resistance and review the rationale and clinical evidence for various PARPi combinations including combinations with chemotherapy, immunotherapy, and targeted therapies. We also highlight emerging PARPi combinations with promising preclinical evidence.

## Introduction

Tumorigenesis is characterised by the hallmark of genomic instability resulting in the accumulation of molecular aberrancies that contribute to the development, progression, and resistance of cancer cells. However, the rapid accumulation of DNA damage in cancer cells leads to increased dependency on specific DNA repair pathways to respond to otherwise potentially cytotoxic DNA damage. The most common type of DNA damage are single-stranded breaks (SSBs) whereas their counterpart, double-stranded breaks (DSBs), are the more lethal form of DNA damage. A critical component of SSB repair, poly (ADP-ribose) polymerase (PARP) remodels chromatin to recruit DNA repair complexes and enables the progression of stalled replication forks [1]. On the other hand, DSBs are repaired by two main pathways: error-prone nonhomologous end joining (NHEJ), or the high-fidelity homologous recombination (HR) repair pathway, which utilises replicated sister DNA as a template [2]. The latter relies on several key proteins including BRCA1/BRCA2 and RAD51 among others [3].

The current generation of PARP inhibitors (PARPi) competitively binds the NAD+ binding moiety on PARP1 and 2, which leads to the induction of DSBs which in turn rely on HR for accurate repair. The concept of PARP trapping, whereby PARP inhibition prevents dissociation of PARPs from DNA, preventing the access of repair proteins and increasing DSBs, has been implicated as a mechanism of action [4]. Indeed, it was elucidated that PARP inhibition was fatal in BRCA1 and BRCA2-deficient cells [5, 6]. This discovery served as a proof-of-concept for clinical approaches and ushered in a new era of cancer-directed therapy. Clinical development of PARPis was heralded by the arrival of olaparib, which was the first to obtain regulatory approval in 2014 as a maintenance strategy in patients with recurrent ovarian cancer [7]. Following the approval of olaparib, various other PARPis including rucaparib, niraparib, and talazoparib have since obtained regulatory approval for various indications in patients with ovarian cancer, breast cancer, prostate cancer, and pancreatic cancer [8].

While PARPis have successfully shifted treatment paradigms in tumours harbouring HR deficiency (HRD), most patients eventually experience resistance attributed to the restoration of homologous recombination repair, amongst other diverse mechanisms [9]. Therefore, it is possible that combination strategies co-targeting pathways that contribute to these described mechanisms of PARPi resistance could result in a deeper and more durable response. In this review, we will discuss current evidence surrounding mechanisms of PARPi resistance and rational PARPi combinations that have been proposed, with a focus on strategies currently in clinical development.

## Mechanisms of PARPi resistance

While PARPis have improved outcomes for a variety of malignancies harbouring HRD, a significant proportion of HRD tumours are innately resistant to PARPi, and those with initial disease control eventually experience disease progression due to the development of a variety of resistance mechanisms. While the mechanisms of innate resistance are poorly understood, several acquired resistance mechanisms have been described.

### Restoration of functional homologous recombination repair

Restoration of at least partial homologous recombination repair function is one of the most well-described mechanisms of resistance that may develop after exposure to platinum therapy or PARP inhibition [10, 11]. Secondary reversion mutations that enable restoration of the HR gene’s open reading frame, allowing for complete transcription of the homologous repair genes, are a well-described mechanism of HR restoration [12]. Reversion mutations have been clinically identified in numerous PARPi-treated tumours and detection in circulating tumour DNA has been associated with treatment resistance or reduced response [12–14]. Reversion mutations have been reported in circulating tumour DNA of up to 39% of patients with *BRCA1/*2-mutated prostate cancer after progression on rucaparib, and approximately 24% of patients with ovarian cancer after treatment with platinum chemotherapy and PARPi [14, 15]. When a *BRCA*1/2 reversion mutation was identified in pretreatment samples from patients with ovarian cancer receiving rucaparib, the median progression-free survival (PFS) was noted to be shorter compared with those without reversion mutations (1.8 months versus 9 months) [14]. Further studies which longitudinally investigate for the development of HR gene (e.g.: *BRCA1/2, RAD51C/D, PALB2)* reversion mutations through a sequential tumour or liquid biopsies are required to comprehensively inform the real-world incidence, timing of development, and impact of such mutations on patient outcomes in clinical settings.

Epigenetic silencing of *BRCA1* or *RAD51C* is a mechanism of homologous recombination deficiency in up to 5–30% of breast and ovarian cancers, with silencing of all *BRCA* copies (homozygous methylation) associated with improved PFS in response to rucaparib compared to *BRCA1/2*-intact cases [16]. However, acquired demethylation of epigenetically silenced *BRCA1* and *RAD51C* promotors has been shown to restore mRNA expression [16–19]. Indeed, among patients with *BRCA* wild-type ovarian cancer with high methylation levels in an archival sample (*n* = 17), none of the patients with methylation loss at enrollment responded to rucaparib versus 38% of patients with maintained methylation [20]. Methylation loss leading to restoration of functional BRCA1/2 can therefore confer PARPi resistance.

Thirdly, alternative mRNA splicing can induce hypomorphic BRCA isoforms which restore HR function. Cancer cell lines and tumours harbouring mutations in exon 11 of *BRCA1* express a BRCA1-Δ11q splice variant lacking the majority of exon 11 but retains partial BRCA activity, mediating PARPi resistance [21]. Similarly, expression of RING domain-deficient BRCA1 (Rdd-BRCA1) has been observed in mammary tumours of mice carrying the BRCA^185stop^ founder mutation which does not require interaction with BARD1 for protein stability, enabling normal HR function in the presence of PARP inhibition [22]. Development of hypomorphic BRCA1 isoforms has been described in patient-derived tumour xenografts (PDXs) from patients with germline *BRCA1/*2-mutant breast cancer resistant to PARPi [23].

### BRCA1/2-independent restoration of HR

In the absence of functional BRCA1/2, other pathways involved in DNA damage response such as those that regulate DNA end resection can be altered in response to PARPi. One of the best-studied mechanisms, inactivating mutations in the *TP53BP1* gene encoding the 53BP1 protein has been implicated in maintaining genetic stability in the setting of BRCA1 loss [24]. 53BP1 in conjunction with the Shieldin complex (REV7, SHLD1, SHLD2 and SHLD3) favours NHEJ by counteracting DSB end resection, which creates the DNA substrate required for HR [25]. Therefore, inactivation of the 53BP1-Shieldin pathway in BRCA1-deficient tumours confers PARPi resistance by restoring DNA end resection and homologous repair of DSBs [23, 26]. Loss of 53BP1 and reduced SHLD1/2 expression has been identified in patient-derived tumour models of PARPi-resistant breast cancer [23, 27]. Among PDXs from patients with germline *BRCA1/*2-mutated breast cancer, 2 of 9 PARPi-resistant models had somatic mutations in *TP53BP1* and exposure to olaparib in PDX models correlated with reduced mRNA expression of SHLD1 and SHLD2 [23, 27]. In addition to HR and NHEJ, DSBs can also be repaired by alternative end joining (TMEJ), mediated by DNA Polymerase Theta (Polθ), and is relied upon particularly in the absence of NHEJ factors such as 53BP1 or members of the Shieldin complex [28].

### DNA replication fork protection

BRCA1/2 are important for DNA replication fork protection, preventing the degradation of stalled replication forks by DNA nucleases [29]. In the absence of BRCA1/2, MRE11 and MUS81 erode replication fork ends, resulting in fork collapse [30]. In BRCA2-deficient cell lines, loss of MLL3/4 complex protein PTIP was associated with decreased MRE11 association with the chromatin which lead to replication fork stability and resistance to PARPi [31]. Alterations such as loss of the nucleosome remodelling factor CHD4 was also shown to decrease recruitment of MRE11 and was present in resistant *BRCA2*-mutant cells [30, 32]. An independent pathway involved in fork degradation, the *EZH2/MUS81* axis, promotes resistance to rucaparib and cisplatin in BRCA2-deficient ovarian cancer cells when downregulated [33].

Ataxia telangiectasia and Rad3-related (ATR) kinase and downstream activation of CHK1 kinase via phosphorylation leads to inhibition of cell cycle progression and restoration of stalled replication forks [34, 35]. In BRCA1-deficient, PARPi-resistant cells, RAD51 loading to DNA DSBs and stalled replication forks occurred in an ATR-dependent fashion [34]. SLFN11 has been identified as a key protein recruited in replication stress that induces irreversible replication block and enhances sensitivity to DNA-damaging agents; and its loss has been associated with PARPi resistance and increased ATR/CHK1 pathway reliance [36]. Expression of SLFN11 has been associated with improved response to DNA-damaging agents in ovarian cancer and small cell lung cancer, and enhancing expression of SLFN11 is a potential strategy under investigation to sensitise tumours to PARPi and other DNA-damaging agents [37].

### Diminishing PARP1 trapping

Alterations in the target of PARPi that reduce binding to PARP1 or alter the function of PARP1 have been uncovered as a mechanism of resistance. PARP inhibition impairs DNA replication by generating PARP-DNA adducts; however, downregulation of PARP1 or alterations in the DNA-binding domains of PARP1 render inhibitors of the PARP enzyme ineffective for PARP trapping [38]. Described mutations in PARP1, such as p.R591C in PARPi-resistant tumour samples, were linked to a diminished PARP1 trapping activity on DNA [38]. Similar to other described resistance mechanisms to PARPi, the incidence of acquired or existing PARP1 mutations in tumours remains poorly defined. The PAR glycohydrolase (PARG) enzyme is a component of PARP1 trapping which counterbalances the activity of PARP1 by catabolizing PAR chains, the product of PARP activity. It was shown in *BRCA2*-mutated mouse mammary tumours that loss of PARG expression enables PARylation despite PARP inhibition, which can contribute to resistance [39].

### PARPi efflux

Increased drug efflux has been identified as a well-described mechanism of resistance to PARPi. Mutations that result in overexpression of *ABCB1* increase transcription of the drug efflux pump MDR1 (P-glycoprotein), and were found in tissue samples from PARPi-treated breast and ovarian cancers [40]. Indeed, *ABCB1* expression was shown to be correlated with resistance to olaparib and rucaparib in ovarian cancer cell lines [41]. In vitro treatment with P-glycoprotein inhibitors has demonstrated the ability to prevent export of PARPi; however, clinical development of such agents has not been successful, likely in part due to the predominance of other resistance mechanisms as well as the toxicity and lack of specificity of these inhibitors [42].

## Rational PARPi combinations under investigation

PARPi combinations are an active area of investigation given their potential to overcome various mechanisms of PARPi resistance. Initial combinations evaluated PARPi in combination with DNA-damaging chemotherapy for synergy, however the development of these combinations has been limited by overlapping toxicity such as myelosuppression. Newer approaches such as antibody-drug conjugates (ADCs) are being evaluated to capitalise on potential synergy with less overlapping toxicity. Other combinations generally fall into a few distinct categories: agents that induce an HRD-like phenotype by inhibiting pathways that promote HR and agents that target alternative DDR pathways which are relied upon during replication stress in response to PARPi. The former includes targeting of commonly upregulated cancer pathways such as PI3K and RAS signalling while the latter includes cell cycle checkpoints such as ATR and WEE1-kinase. Another promising approach, immunotherapy combinations have potential synergistic activity with limited overlapping toxicity.

## PARPi and targeted therapies

### PARPi and PI3K/AKT/MTOR inhibition

**Fig. 1:**
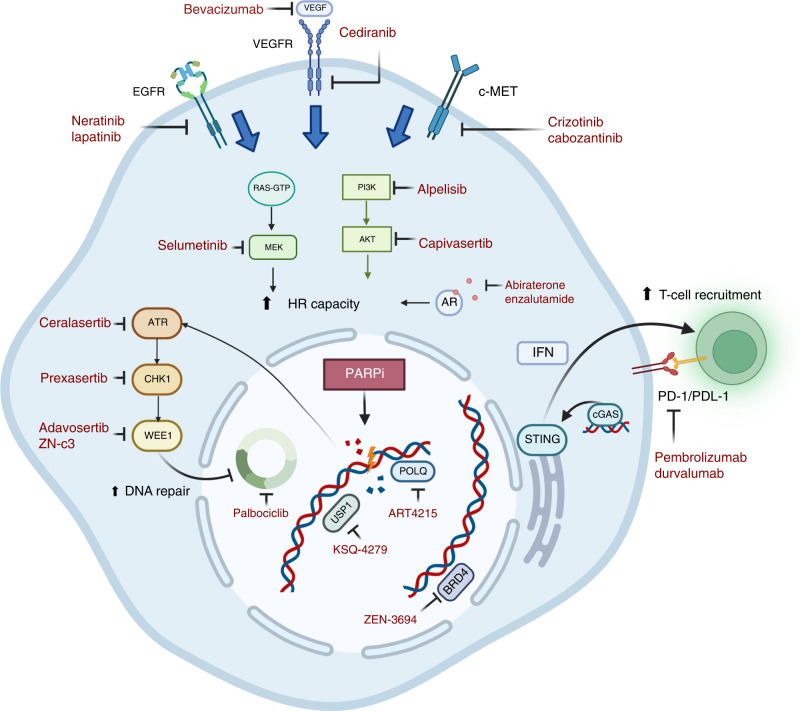
Emerging targets for rational combinations with PARP inhibitors. The figure displays various pathways that modulate response to DNA damage and are identified as key targets in combination with PARP inhibitors. The receptor tyrosine kinase cascade mediated by the EGFR, VEGF and c-MET receptors with downstream effectors such as the RAS/MEK and PI3K/AKT pathways are hypothesised to increase homologous repair capacity. Pathways and proteins involved in cell cycle progression such as ATR and WEE1 play an integral role in response to DNA damage in addition to emerging targets USP1 and POLQ. DNA damage mediated by PARP inhibitors is hypothesised to increase T-cell recruitment via activation of the cGAS-STING pathway.

Effective blockade of the PI3K/mTOR pathway has been hypothesised to induce HRD via various mechanisms including suppression of DNA double-strand repair protein SUV39H1 and suppression of homologous recombination repair gene expression [43]. Combinations of PI3K inhibition with PARPi have been investigated in preclinical studies and early-phase clinical trials as an approach to extend the benefit of PARPi to tumours lacking HRD (Fig. 1). In one study, PI3K inhibition in BRCA-proficient triple-negative breast cancer PDX models resulted in ERK activation and downstream activation of ERK-activated transcription factor (ETS1) to downregulate expression of *BRCA1/2* [44]. To date, early-phase trials evaluating the combination of olaparib with the pan-PI3K inhibitor buparlisib have demonstrated preliminary efficacy (ORR 30%) in patients with breast and ovarian cancer regardless of *BRCA1/*2-mutation status, although at the cost of adverse events such as mood disturbance requiring multiple dose reductions of buparlisib [45]. Similarly, the PARPi olaparib has been combined with the α-selective PI3K inhibitor alpelisib in a Phase 1b study of recurrent triple-negative or germline *BRCA1* and BRCA2-mutated breast cancer [46]. In this study, 3 of 17 evaluable patients achieved a partial response, none of whom harboured a germline *BRCA1/2* mutation [46]. In patients with recurrent and predominantly platinum-resistant ovarian cancer, ORR was 36% with responses seen among those without germline HRR mutations [47]. These early studies appear to indicate that PI3K pathway inhibition confers dysfunctional HR repair, which sensitise tumours to PARPi; and is being further evaluated against chemotherapy in platinum-resistant/refractor ovarian cancer without germline *BRCA1/*2 mutation in the EPIK-O trial (NCT04729387). Inhibition of the downstream target Akt has been identified as an alternative PI3K pathway target in combination with PARPi. In a Phase 1 study enrolling patients with recurrent endometrial, breast, and ovarian cancer to receive Akt inhibitor capivasertib and olaparib, 6 of 38 evaluable patients (19%) achieved a partial response and there were fewer dose-limiting toxicities [48]. There were also several identified molecular correlates of response including increased activation of DNA damage checkpoints and decreased mTOR activation, while receptor tyrosine kinase activity and RAS-MAPK pathway activity were associated with resistance. The combination was also assessed in a Phase 1 trial enrolling patients with *BRCA1/2*, HRD-altered, and/or *PI3KCA*-mutated cancers; 14 (25%) patients achieved a partial response and the combination was safe [49]. The most common adverse events included gastrointestinal toxicities with a grade 3 dose-limiting toxicity of rash noted at higher doses of capivasertib. These studies indicate the feasibility and activity of PARP-PI3K pathway co-targeting, yet further data regarding predictive biomarkers of response and how the addition of PI3K pathway targeting affects acquired resistance mechanisms are still awaited from ongoing studies.

### PARPi and antiangiogenic therapies

The vascular endothelial growth factor (VEGF) protein family consists of growth factors that promote angiogenesis in the context of hypoxia. Preclinical studies have demonstrated a link between PARP1 and angiogenesis, with PARP1-knockout in mouse models of skin cancer resulting in decreased HIF-1α and consequently reduced angiogenesis [50]. At the same time, induction of hypoxia via inhibition of VEGF is associated with downregulation *BRCA1/2* and *RAD51*, in *BRCA1/2* wild-type ovarian cancer cell lines [51–53]. In ovarian cancer cell lines, it was shown that cedinarib-induced hypoxia activated the p130/E2F4 complex that binds to E2F consensus sequences in the promoters of HRR genes to decrease transcription [53]. However, others were unable to replicate the HRR-altering effects of cedinarib in orthotopic ovarian cancer PDXs, despite noting synergy of the combination [52]. Consequently, the rational combination of antiangiogenics with PARPi have been explored in clinical studies, primarily in the setting of ovarian cancer. The Phase 3 PAOLA-1 study evaluated the combination of PARPi and antiangiogenic treatment with bevacizumab as a first-line maintenance strategy after initial platinum-based chemotherapy in advanced ovarian cancer [54]. The trial met its primary endpoint of improved median PFS for olaparib plus bevacizumab versus bevacizumab alone, which has led to the regulatory approval of this combination. Combinations of PARPi and bevacizumab or oral VEGF tyrosine kinase inhibitor, cediranib, has also been explored extensively in recurrent ovarian cancer as a chemotherapy-free alternative in the platinum-sensitive and resistant disease setting [55–58] (Table 1). The combination of niraparib and bevacizumab was evaluated in patients with platinum-sensitive recurrent ovarian cancer where it demonstrated superior PFS compared to niraparib alone (11.9 months vs 5.5 months) [55]. NIRVANA-1 (NCT05183984) and NIRVANA-R (NCT04734665) are assessing the combination in patients after complete cytoreduction and in patients with prior PARPi exposure, respectively.

**Table 1:** Efficacy data for selected studies evaluating PARP inhibitor combinations in advanced solid tumours.

Drug class	Reference (phase)	Treatment	Study population	Overall efficacy
Platinum chemotherapy	Ramalingam et al. (Phase 3) [141]	Carboplatin/paclitaxel +/− veliparib	Previously untreated advanced squamous cell lung cancer (*n* = 970)	Median PFS: 5.6 mo in both groups (carboplatin/paclitaxel with or without veliparib)
	O’Reilly et al. (Phase 2) [142]	Gemcitabine/cisplatin +/− veliparib	Advanced pancreatic adenocarcinoma, germline *BRCA*1/2 or *PALB2* mutation (*n* = 50)	Median PFS: 10.1 mo (chemo + veliparib) vs 9.7 mo (chemo + placebo) ORR: 20/27 (74%) with veliparib vs 15/23 (65%) with placebo
	Diéras et al. (Phase 3) [143]	Carboplatin/paclitaxel +/− veliparib	*BRCA1/2*-mutated advanced breast cancer (*n* = 513)	Median PFS: 14.5 mo (chemo + veliparib) and 12.6 mo (chemo)
DNA alkylators	Pietanza et al. (Phase 2) [144]	Temozolomide +/− veliparib	Previously treated SCLC (*n* = 104)	PFS at 4 months: 36% (TMZ/veliparib) and 27% (TMZ/placebo) ORR: 19/49 (39%) for TMZ/veliparib and 6/44 (14%) for TMZ/placebo
	Plummer et al. (Phase 2) [136]	Temozolomide + rucaparib	Chemotherapy naive metastatic melanoma (*n* = 46)	ORR: 8/46 (17%)
	Pishvaian et al. (Phase 2) [131]	Temozolomide + velaparib	Treatment-refractory metastatic colorectal cancer (*n* = 50)	ORR: 2/50 (4%)
	Farago et al. (Phase 2) [132]	Temozolomide + olaparib	Previously treated small cell lung cancer (*n* = 48)	ORR: 20/48 (42%)
	Xu et al. (Phase 2) [133]	Temozolomide + olaparib	Metastatic breast cancer (*n* = 62)	ORR: 7/62 (12%)
Topoisomerase Inhibitors	LoRusso et al. (Phase 1) [138]	Irinotecan + veliparib	Previously treated advanced solid tumours (*n* = 31)	ORR: 6/31 (19%)
	Gorbunova et al. (Phase 2) [145]	5-Fluorouracil/irinotecan +/− veliparib	Untreated metastatic colorectal cancer (*n* = 130)	Median PFS: 12 mo (FOLFIRI/veliparib) vs 11 mo (FOLFIRI)
	Yap et al. (Phase 1) [140]	Sacituzumab + rucaparib	Advanced solid tumours with or without mutations in HR genes (*n* = 6)	ORR: 3/6 (50%)
Anti-VEGF	Liu et al. (Phase 3) [56]	Olaparib +/− cediranib versus platinum-based chemotherapy	Recurrent platinum-sensitive ovarian cancer (*n* = 565)	Median PFS: 10.3 mo (chemo), 8.2 mo (olaparib), and 10.4 mo (olaparib/cediranib)
	Mirza et al. (Phase 2) [55]	Niraparib +/− bevacizumab	Recurrent platinum-sensitive ovarian cancer (*n* = 97)	Median PFS: 11.0 mo (niraparib/bevacizumab) and 5.5 mo (niraparib)
	Colombo et al. (Phase 2) [57]	Paclitaxel vs cediranib + olaparib (continuous or intermittent)	Recurrent platinum-resistant ovarian cancer (*n* = 123)	Median PFS: 3.1 mo (paclitaxel), 5.6 mo (continuous cediranib + olaparib), 3.8 mo (intermittent cediranib + olaparib)
	Lheureux et al. (Phase 2) [58]	Cediranib + olaparib	Treatment-refractory ovarian cancer with progression on PARPi (*n* = 34)	ORR: 3/34 (9%)
	Lee et al. (Phase 2) [59]	Cediranib + Olaparib	HRD-positive platinum-resistant ovarian cancer (*n* = 16)	ORR: 8/16 (50%)
	Ray-Coquard et al. (Phase 3) [54]	Bevacizumab +/− olaparib maintenance	Ovarian cancer responsive to first-line platinum chemotherapy (*n* = 806)	Median PFS: 22.1 mo (bevacizumab/olaparib) vs 16.6 mo (bevacizumab)
	Hardesty et al. (Phase 2) [146]	Bevacizumab + niraparib maintenance	Ovarian cancer responsive to first-line platinum chemotherapy (*n* = 105)	Median PFS: 19.6 months
	Cecchini et al. (Phase 1/2) [62]	Ramucirumab + olaparib	Refractory metastatic gastric or GEJ adenocarcinoma (*n* = 46)	ORR: 5/46 (11%)
PI3K/AKT inhibitor	Batalini et al. (Phase 1) [46]	Alpelisib + olaparib	Advanced triple-negative breast cancer and recurrent breast cancer with *gBRCA1/2* mutation (*n* = 17)	ORR: 3/17 (18%)
	Konstantinopoulos et al. (Phase 1) [47]	Alpelisib + olaparib	Recurrent ovarian cancer with g*BRCA1/2* mutation (*n* = 28)	ORR: 10/28 (36%)
	Yap et al. (Phase 1) [49]	Capivasertib + olaparib	Advanced solid tumours and *gBRCA1/2* mutation or *BRCA1/2* wild type with DDR or PI3K-AKT pathway alterations (*n* = 56)	ORR: 19/56 (34%)
	Westin et al. (Phase 1) [48]	Capivasertib + olaparib	Recurrent endometrial, ovarian, and triple-negative breast cancer (*n* = 32)	ORR: 6/32 (19%)
EGFR inhibitor	Stringer-Reasor et al. (Phase 1) [74]	Veliparib + lapatanib	Metastatic triple-negative breast cancer without *gBRCA1/2* mutation (*n* = 17)	ORR: 4/17 (24%)
MEK inhibitor	Kurnit et al. (Phase 1) [65]	Selumetinib + olaparib	Advanced solid tumours with RAS pathway alterations (*n* = 12)	ORR: 2/12 (17%)
BET inhibitor	Aftimos et al. (Phase 1b/2) [68]	ZEN-3694 + talazoparib	Metastatic triple-negative breast cancer without *gBRCA1/2* mutations (*n* = 50)	ORR: 11/50 (22%)
Immunotherapy	Lee et al. (Phase 1) [83]	Durvalumab + olaparib	Recurrent or metastatic ovarian, breast, cervical, or endometrial cancer (*n* = 12)	ORR: 2/12 (17%)
	Domchek et al, Bang et al., Thomas et al. (Phase 1/2 MEDIOLA) [84, 85, 91]	Durvalumab + olaparib	Advanced germline BRCA-mutated breast cancer and ovarian cancer; metastatic gastric cancer and relapsed SCLC (*n* = 88)	ORR: 4/39 (10%) in gastric cancer; 19/30 (63%) in *gBRCA1/2*-mutated breast cancer; 2/19 (11%) in SCLC
	Konstantinopoulos et al., Vinayak et al. (Phase 1/2 TOPACIO/KEYNOTE-162) [87, 92]	Niraparib + pembrolizumab	Recurrent platinum-resistant ovarian cancer and advanced TNBC (*n* = 115)	ORR: 10/60 (18%) in ovarian cancer; 10/55 (18%) in TNBC
	Reiss et al. (Phase 1b/2) [93]	Niraparib + ipilimumab or niraparib + nivolumab maintenance after platinum chemotherapy	Advanced platinum-sensitive pancreatic cancer (*n* = 91)	6-month PFS: 21% with niraparib + nivolumab and 60% with niraparib + ipilimumab
	Friedlander et al. (Phase 1) [82]	Pamiparib + tislelizumab	Advanced solid tumours (*n* = 49)	ORR: 10/49 (20%)
	Yap et al. (Phase 1) [94]	Dostarlimab with niraparib, carboplatin-paclitaxel, with or without bevacizumab	Advanced solid tumours (*n* = 35)	ORR: 4/22 (18.2%) with niraparib and 4/13 (30.8%) with niraparib + bevacizumab
ATR inhibitor	Shah et al. (Phase 2) [102]	Ceralasertib + olaparib	Recurrent platinum-resistant, PARPi-naive ovarian cancer (*n* = 12)	ORR: 0/12 (0%)
	Mahdi et al. (Phase 2) [147]	Ceralasertib + olaparib	Advanced solid tumours with deleterious germline or somatic alterations in HR genes (*n* = 25)	ORR: 2/25 (8.3%)
CHK1 inhibitor	Do et al. (Phase 1) [148]	Prexasertib + olaparib	Advanced solid tumours, ovarian cancer with *BRCA1/2* mutation. Previous PARPi allowed (*n* = 18)	ORR: 4/18 (22%) in patients with *BRCA1/2*-mutant, PARP inhibitor–resistant ovarian cancer
WEE1 inhibitor	Westin et al. (Phase 2) [111]	Adavosertib +/− olaparib	Recurrent ovarian, fallopian tube, or primary peritoneal cancer with documented progression on PARPi; 48% had germline or somatic *BRCA1/2* mutation (*n* = 35)	ORR: 10/35 (29%) with adavosertib + olaparib vs 8/35 (23%) adavosertib
	Yap et al. (Phase 1) [113]	Sequential adavosertib and olaparib	Advanced cancer with actionable DDR variants (*n* = 12)	ORR: 3/12 (25%)
Antiandrogen	Clarke et al. (Phase 3) [123]	Abirateraone +/− olaparib	Metastatic castration-resistant prostate cancer (*n* = 796)	Radiographic PFS: 24.8 mo (abiraterone/olaparib) vs 16.6 mo (abiraterone)
	Chi et al. (Phase 3) [124]	Abirateraone +/− niraparib	Metastatic castration-resistant prostate cancer (*n* = 423)	Radiographic PFS in HR-deficient mCRPC: 16.5 mo (abiraterone/niraparib) vs 13.7 mo (abiraterone)

*PFS* progression-free survival, *ORR* overall response rate, *TMZ* temozolomide, *mo* months, *chemo* chemotherapy, *gBRCA1/2* germline BRCA1 and BRCA2, *SCLC* small cell lung cancer, *DDR*   DNA damage response, *TNBC* triple-negative breast cancer, *HR* homologous repair, *PARPi*  PARP inhibitor, *mCRPC* metastatic castration-resistant prostate cancer.

While the combination of cediranib with olaparib has demonstrated activity as a chemo-free regimen for recurrent platinum-sensitive ovarian cancer, the recent Phase 3 NRG-GY004 trial in patients with platinum-sensitive ovarian cancer did not demonstrate an improvement in PFS with the combinations of olaparib plus cediranib versus platinum-based chemotherapy [56]. Nevertheless, the subgroup of patients with germline *BRCA1/2* mutation appeared to benefit most with a PFS hazard ratio versus chemotherapy of 0.55 (95% CI, 0.32–0.94) for olaparib/cediranib and 0.63 (95% CI, 0.37–1.07) for olaparib in this PARPi-naive population (Table 1) [56]. Several other Phase 2 trials have evaluated the olaparib plus cediranib combination in platinum-resistant ovarian cancer with promising activity observed [57, 59, 60] (Table 1). In the AMBITION study HRD-positive patients with heavily pre-treated platinum-resistant ovarian cancer were randomised to receive either olaparib and cediranib versus olaparib and durvalumab with response rates of (8/16) 50% and (6/14) 43% respectively [59]. In both the BAROCCO and OCTOVA trials, the median progression-free survival for the olaparib plus cediranib combination (dosed continuously) trended towards improvement compared with paclitaxel alone [57, 60]. These benefits were observed irrespective of *BRCA1/2* genomic status, or prior antiangiogenic or PARPi use, in the OCTOVA trial [60]. In the EVOLVE study, patients with progression on PARPi for recurrent ovarian cancer received olaparib and cedinarib; in this context, only three objective responses (9%) were noted, with several patients having developed reversion mutations in HR genes as proposed mechanism of resistance [58]. Further Phase 3 ongoing trials evaluating this combination include ICON 9 (NCT03278717) and NRG-GY005 (NCT02502266) in patients with the platinum-resistant disease and in the maintenance setting for platinum-sensitive recurrent ovarian cancer, respectively.

There have been relatively limited studies and successes in non-ovarian cancer pathologies such as pancreatic cancer, where antiangiogenics and PARPi have limited activity in HR proficient disease [61]. Ongoing trials are assessing the combination of various antiangiogenic agents with PARPi in gastric, endometrial (NCT03570437), cervical (NCT04487587), breast (NCT04090567) and prostate cancers (NCT02893917) [62]. The PARPi plus antiangiogenic strategy is promising, but integrating such combinations into treatment paradigms needs further data to clarify their role, including molecular correlates of response and impact on PARPi-resistance mechanisms.

### PARPi and RAS/RAF/MEK pathway targeting

Promising preclinical data have demonstrated synergism between MEK inhibition and PARP inhibition in vitro and in vivo [63, 64]. MEK inhibition was shown to decrease expression of components of the homologous recombination DNA repair pathway and decrease HR repair capacity, in part by diminishing the expression of FOXO3a, to enable synergy with PARPi, which has led to the evaluation of MEKi and PARPi combinations in the clinic [63]. In an ongoing trial evaluating MEK inhibitor selumetinib in combination with olaparib in primarily K*RAS*-mutated tumours, 2 of 14 patients had a confirmed partial response, and the combination was safe and tolerable [65].

### PARPi and BET inhibition

The BET bromodomain family of proteins is thought to promote oncogenesis and genes related to homologous repair [66]. Preclinical data demonstrated that inhibition of BET protein BRD4 resulted in a homologous repair-deficient phenotype, which was associated with PARPi sensitivity in vitro and in vivo regardless of *BRCA1/2* or *RAS/RAF* status, suggesting that the combination could have activity outside of HRD-positive tumours. This was mechanistically shown to be mediated by the downregulation of CtIP which is an important component of DSB repair [67]. An early-phase trial assessing the BET inhibitor ZEN-3694 with talazoparib in patients with germline *BRCA1/2* wild-type tumours demonstrated preliminary efficacy with ORR of 22% and clinical benefit rate of 35% among 51 evaluable patients, with thrombocytopenia reported as the most common adverse event [68]. An ongoing trial is evaluating the combination in patients with prior PARPi exposure or *KRAS*-mutated tumours (NCT05327010).

### PARPi and c-MET inhibition

c-MET has been shown to be a key mediator of PARP inhibition by phosphorylating PARP1 at Tyr90 which increases enzymatic activity of PARP1 and decreases binding of PARPi [69]. The c-MET inhibitor crizotinib demonstrated synergy with PARP inhibition in preclinical breast, lung and ovarian cancer models [69, 70]. A sequential combination of crizotinib and a PARPi resulted in activation of ATM/CHK2 and inhibition of c-Met pathways, contributing to a decrease in RAD51 levels and induced apoptotic cell death in ovarian cancer cell lines [70]. There are several ongoing trials evaluating the combination of PARPi and c-MET tyrosine kinase inhibitors including crizotinib (NCT04693468) and cabozantinib (NCT03425201, NCT05038839) in advanced solid tumours.

### PARPi and EGFR/HER2 targeting

Overexpression of the epidermal growth factor receptor (EGFR) induces tumour growth and is involved in regulating DNA damage response in preclinical studies [71]. Preclinical work in hepatocellular carcinoma and breast cancer reveal that interactions between c-MET and EGFR result in the phosphorylation of PARP1 to mediate resistance [72, 73]. Lapatinib was evaluated in combination with veliparib in a Phase 1 study enrolling patients with *BRCA1/2* wild-type metastatic TNBC; of 17 evaluable patients, 4 patients achieved a partial response [74]. The combination of pan-EGFR inhibitor neratinib was synergistic with niraparib in ovarian cancer cell lines as well, leading to the ongoing clinical trial evaluating the combination in patients with platinum-resistant, *BRCA1/2* wild-type ovarian cancer (NCT04502602) [75]. Other studies including the combination of niraparib with osimertinib (NCT 03891615), trastuzumab (NCT 03368729), and trastuzumab deruxtecan (NCT04585958) in EGFR-positive lung and HER2-positive cancers are underway.

## PARPi and immune checkpoint blockade combinations

Anti-PD(L)1 and -CTLA4 Immune checkpoint blockade has rapidly altered the field of oncology, resulting in multiple approvals across tumour types and indications [76]. Efforts have been made to enhance the effectiveness of immunotherapy and broaden indications for use through various combination strategies, especially for tumour subtypes that are conventionally thought to be immunotherapy-resistant [77]. Mechanistically, the combination of PARP inhibition and immunotherapy has been of interest due to synergistic effects noted in preclinical models [78]. PARPi induces DSBs resulting in generation of double-strand DNA (dsDNA) that, through cyclic guanosine monophosphate–adenosine monophosphate synthase (cGAS) binding, results in activation of the stimulator of interferon genes (STING) pathway [78]. The cGAS/STING pathway upregulates chemokines CCL5 and CXCL10 leading to the recruitment of CD8 T cells responsible for anti-tumour immunity [79, 80]. PARPi was also found to coincide with upregulation of immune checkpoint PDL-1 [80]. In preclinical models, it was shown that PARP inhibition and anti-PD1 therapy was synergistic regardless of *BRCA1/2* mutation in colon, breast and ovarian experimental models in vitro and in vivo [78, 81]. While this combination does not directly address mechanisms of PARPi resistance, it is hypothesised to expand the benefit of PARPi beyond tumours harbouring a ‘*BRCA*ness‘ or HRD phenotype, with the benefit of minimal overlapping toxicity from both drug classes.

Promising preclinical data have prompted the evaluation of PARPi and anti-PD(L)1 combinations in the clinic. Phase I trials demonstrated overall safety profile and preliminary efficacy in a population of patients with advanced solid tumours, with no new adverse events with the combination compared with either monotherapy [82, 83]. The MEDIOLA trial is a Phase II basket study assessing the efficacy and safety of anti-PD1 agent durvalumab and olaparib in patients with solid tumours and a germline *BRCA1/2* mutation [84, 85]. The treatment consisted of olaparib alone for 4 weeks followed by a combination of olaparib and durvalumab until disease progression. Among patients with platinum-sensitive recurrent ovarian cancer who received at least one line of platinum therapy, ORR was 71.9%, with a total of 7 complete responses and the median PFS was 11.1 months. Among the 34 enrolled patients with HER2-negative breast cancer, ORR was 56% including 1 patient with complete response, and median PFS was 6.7 months [84, 86]. Anaemia, neutropenia, and pancreatitis were the most common grade 3 or 4 adverse events, consistent with previous reports from studies assessing PARPi or immunotherapy.

The TOPACIO/KEYNOTE-162 Phase I/II study evaluated the combination of niraparib and pembrolizumab in patients with recurrent platinum-resistant ovarian cancer irrespective of *BRCA1/*2-mutation status [87]. Among 60 patients with evaluable response, ORR was 18% with disease control rate of 65%, including three patients with complete response [87]. Notably, the response rate with the combination of niraparib and pembrolizumab was 19% among patients with *BRCA1/2* wild-type tumours, which compares favourably to previously reported ORR of less than 10% with either agent as a monotherapy in this population [88, 89]. Overall, these data suggest that the combination of PARPi and anti-PD1 therapy is safe and effective, though further data is needed to delineate which populations benefit most from the combination. There are several ongoing trials assessing the combination in non-breast/ovarian histologies, and others evaluating triplet combinations of PARPi, immune checkpoint blockade and a third targeted agent such as antiangiogenic (NCT04361370) or AKT inhibitors (NCT03772561) [90–94]. Translational studies have attempted to characterise features associated with response to the PARP inhibitor and immune checkpoint inhibitor combination [95, 96]. Among patients with ovarian cancer enrolled in the TOPACIO/KEYNOT-162 study evaluating niraparib and pembrolizumab, tumours from responders typically had features of HRD (mutational signature 3) and increased presence of exhausted CD8 + T cells (immune score) versus non-responders [95]. Biopsies from patients receiving durvalumab and olaparib in the MEDIOLA trial revealed increased STING and IFN-1 pathway activity in responders, and lack of increase was associated with resistance to treatment [96].

## PARPi and DNA damage response inhibitors

Upregulation of alternative pathways involved in DNA damage and replication stress are implicated as mechanisms for PARPi resistance. Therapeutic approaches targeting proteins involved in replication stress and DNA repair are therefore being investigated as a potential strategy to overcome PARPi resistance.

### PARPi and ATR inhibitors

One area of investigation has been the combination of PARPi with inhibitors of the ATR kinase, a protein that is involved in the regulation of cellular response to stalled and collapsed replication forks through downstream activation of the CHK1/WEE1 axis [97, 98]. Moreover, ATR and CHK1 act to stabilise and protect stalled replication forks by mediating fork remodelling in cooperation with RAD51, ZRANB3, and SMARCAL1. Inhibition of the ATR/CHK1 pathway disrupts cell cycle progression, leading to chromosomal aberrations, mitotic catastrophe, and apoptosis [99]. Promising preclinical data has led to the development of several ATR inhibitors that have undergone clinical investigation [100]. PARPi have been shown to result in early activation of ATR/CHK1 pathway, and the combination of olaparib with ATR inhibitor ceralasertib synergistically suppressed growth in PDX models of *BRCA1/*2-mutant HGSOC [101]. Importantly, the combination has demonstrated activity in the setting of *BRCA1/2* reversion mutations which is a common mechanism of PARPi resistance [34, 101]. In terms of clinical data, a Phase 2 study of ceralasertib and olaparib conducted in patients with recurrent, epithelial ovarian cancer has reported preliminary efficacy in this population [102]. In 14 enrolled patients with the platinum-resistant disease who were PARPi-naive, the majority of which had no somatic or germline alterations in HR genes, the best response was stable disease (SD), which was achieved in 9 of 12 evaluable patients and median PFS was 4.2 months; patients with germline or somatic HR alterations appeared to derive increased benefit from the combination. Most toxicities were limited grade 1 or 2, and the combination was well-tolerated. Confirmed biomarkers for ATR inhibitors or ATR and PARPis are not fully elucidated although multiple potential genomic biomarkers have been described in preclinical data [103–105]. In vitro models of prostate cancer with ATM loss were sensitive to ATR inhibition which was enhanced with the addition of PARPi [105]. Co-deletion of *RB1* and *RNASEH2B* was associated with PARPi resistance in prostate cancer cell lines due in part to E2F1-induced BRCA2 expression, however, inhibition of ATR was able to overcome this resistance via disruption of E2F1-induced BRCA2 expression [103]. And while *CDK12* loss is not classically sensitive to PARPi, the addition of ATR inhibition sensitised *CDK12-deficient* cells to PARPi [104]. These data highlight the potential for PARPi combinations to overcome intrinsic resistance to PARPi.

### PARPi and WEE-like kinase 1 inhibitors

WEE1 modulates cell cycle progression through the S and G2/M checkpoints via regulation of CDK1 and 2, and inhibition has been shown to promote premature mitotic entry and mitotic catastrophe [106, 107]. Inhibitors of WEE1 have therefore been evaluated as a candidate for combination with PARP inhibition. The combination was shown to be synergistic in PARPi-resistant models across several histologies [108–110]. Moreover, the WEE1 inhibitor adavosertib alone or in combination with olaparib was assessed in a Phase II study enrolling patients with recurrent ovarian cancer with previous progression on PARPi treatment, approximately half with a germline or somatic *BRCA1/2* mutation [111]. Among the 35 patients receiving combination therapy, ORR was 29% versus 23% for adavosertib alone, and the clinical benefit rate was 89%. Due to the overlapping toxicity profile, there was increased myelosuppression and gastrointestinal toxicity from the combination describe the incidence of high grade toxicity [111]. One approach to improve tolerability while maintaining efficacy has been to sequentially dose DDR inhibitors such as WEE1 inhibitors, which was shown to be feasible in ovarian cancer PDX models and on a recently reported Phase I trial [112, 113]. Only 1 of 13 (8%) of patients required a dose reduction for toxicity and disease control was seen despite prior PARPi exposure [113].

### PARPi and CDK4/6 inhibitors

Inhibition of cell cycle progression with cyclin-dependent kinase (CDK) inhibitors have greatly altered the landscape for patients with hormone receptor-positive breast cancer, and rational combination of CDK4/6 inhibitors with PARPis has generated interest due to preclinical data demonstrating synergy in TNBC, ovarian, and neuroendocrine prostate cancer [114–117]. CDK4/6 inhibition was shown to downregulate homologous recombination, sensitising breast and ovarian cancer cell lines to PARP inhibition to inhibit cell growth, particularly in tumours with high levels of MYC expression [117]. It is hypothesised that the synergistic effects from CDK4/6 inhibition are mediated by inhibition of the MYC pathway, which was shown to regulate expression of *BRCA1/2* and *RAD51* [117]. In prostate cancer cell lines, the combination was shown to suppress the p-Rb1-E2F1 signalling axis leading to inhibition of E2F1 gene targets such as *BRCA1/2* and RAD51 among others [116]. On this basis, PARPi and CDK4/6 inhibitor combinations are currently under evaluation including in MYC amplified tumours (NCT04693468).

### PARPi with other novel DDR targets—POLQ inhibitors and USP1 inhibitors

In addition to HR and NHEJ, theta-mediated end joining, also known as microhomology-mediated end joining (MMEJ), is an error-prone escape pathway mediating repair of double-stranded DNA breaks which relies on Polθ (POLQ) [28]. Knockdown of POLQ was shown to be lethal in HRD tumours, due to their increased reliance on the MMEJ pathway [118, 119]. It was demonstrated that inhibition of POLQ inhibition with novobiocin was able to overcome PARPi resistance in cell lines with increased replication fork stability and HR capacity [119]. Though, cell lines with a *BRCA1/2* reversion were not sensitive to POLQ inhibition. An orally bioavailable inhibitor, ART4215, is being evaluated in early-phase clinical trials as a monotherapy or in combination with PARPi (NCT04991480) [28]. USP1 is a deubquitinase which has emerged as a promising target as it plays a key role in stabilisation of the replication fork [120]. Particularly in BRCA1-deficient cells, USP1 prevents the recruitment of TLS polymerases to the fork by preventing the accumulation of monoubiquinated Proliferating cell nuclear antigen (PCNA-Ub) at the replication fork. In the absence of USP1, BRCA1-deficient cells (but not BRCA2-deficient cells) are unable to tolerate elevated PCNA-Ub levels which lead to TLS polymerase accumulation at the fork, replication fork instability, and mitotic catastrophe. USP1 inhibition suppressed cell growth among PARPi-resistant BRCA1-deficient cell lines with restored replication fork stability. A novel USP1 inhibitor KSQ-4279 is currently undergoing evaluation alone and in combination with PARPi in advanced cancers (NCT05240898).

## PARPi and novel hormonal agents

The androgen receptor has been directly implicated in the expression of DNA repair genes such as DNA-dependent protein kinase catalytic subunit (DNA-PKcs), a protein essential for nonhomologous end joining (NHEJ) [121, 122]. Androgen signalling also regulates the expression of XRCC2 and XRCC3, which are important for HR [122]. It has therefore been hypothesised that co-inhibition of PARP and antiandrogens could induce tumour regression independent of the presence of HR alterations. Several Phase 3 trials have read out on combinations of novel hormonal agents with PARPi, with further trials ongoing (Table 1). In the Phase 3 PROpel trial which enrolled patients with metastatic castration-resistant prostate cancer to receive abiraterone plus olaparib versus abiraterone plus placebo, median imaging-based PFS was longer in the combination group (24.8 vs 16.6 months) irrespective of HR status [123]. On the contrary, early results from the Phase 3 MAGNITUDE study assessing the combination of niraparib and abiraterone in a similar patient population revealed a significant benefit with the combination of niraparib and abiraterone in patients with *BRCA1/*2-mutated or HRR-mutated cancer (defined as *ATM, BRCA1, BRCA2, BRIP1, CDK12, CHEK2, FANCA, HDAC2, PALB2* mutations), but not HRR-negative prostate cancer [124]. Longer-term follow-up, translational research, and results from concomitant trials (Table 1) assessing the combination of novel hormonal agents with PARPi are awaited to clarify the value of the combination in patients with metastatic castration-resistant prostate cancer.

## PARPi and chemotherapy

The complementary DNA-damaging effects of PARPi and chemotherapy have provided rationale for exploring their combination (Table 1). While these combinations do not directly address resistance mechanisms to PARPi, they may increase the efficacy and delay the development of resistance compared with PARPi monotherapy. Combinations of PARPis with platinum chemotherapy lead to cross-linking of DNA strands, which prevents DNA replication and transcription, leading to cell cycle arrest, and eventual apoptosis [125]. Yet, significant challenges have been met in the development of PARPi—platinum-based chemotherapy combinations due to overlapping myelosuppression which has limited the feasibility of this strategy. This has led to PARPi being developed as a sequential maintenance strategy after the initial response to platinum-based chemotherapy in advanced ovarian cancer, with several PARPi including olaparib, niraparib, and rucaparib demonstrating feasibility and efficacy over placebo as front-line maintenance therapy [126–128]. Combinations of PARPi with non-platinum chemotherapies may remain feasible. Temozolomide, an orally administered DNA-alkylator, has demonstrated enhanced cytotoxic effects when combined with PARPis in preclinical studies, independent of HR [129, 130]. Temozolomide induces base damage which leads to the formation of PARP-DNA complexes which, when combined with potent PARP-trapping agents, enhances cytotoxicity. This has prompted investigation into trials assessing this combination across various tumour histologies including colorectal cancer, small cell lung cancer, breast cancer, glioblastoma, prostate cancer, and melanoma [131–136]. Generally, the combination of temozolomide and PARP inhibition was shown to be well-tolerated; however, efficacy was modest in most tumour subtypes (Table 1). Topoisomerase inhibitors such as irinotecan fix Top1 onto DNA and generate unrepaired SSBs which are lethal in malignant cells. PARP1 is critical for repair of Top1 cleavage sites in DNA, thus PARP inhibition results in increased generation of SSBs and synergism in preclinical models [137]. The therapeutic efficacy and safety of PARP inhibition and irinotecan have been explored in clinical studies, but reports of increased haematologic and gastrointestinal toxicities have limited their adoption [138]. Antibody-drug conjugates (ADC) have been developed to deliver cytotoxic chemotherapy in a more targeted fashion to target-expressing cancer cells; these have the advantage of wider therapeutic windows and reduced toxicity [139]. Sacituzumab govitecan, a tumour-associated calcium signal transducer 2 (Trop-2) targeting ADC containing the active metabolite of irinotecan (SN-38) has been assessed in an early-phase trial in combination with the PARPi rucaparib. It has so far demonstrated promising clinical activity including patients with prior exposure to PARPis, with less overlapping toxicity than seen in PARPi/irinotecan combinations [140]. The ongoing PETRA trial is assessing PARPi in combination with trastuzumab deruxtecan and Trop-2-targeting ADC datopotamab (NCT04644068).

## Conclusion

PARPis are versatile drugs with established antitumor activity in a number of settings. PARPi resistance is a key challenge that has prompted investigation into numerous rational combinations at various stages of clinical investigation. So far, there are promising clinical responses which have validated preclinical findings for PARPi combination partners across a variety of drug classes. Further work is needed to optimise the therapeutic window of PARPi combinations and identify confirmed predictive biomarkers of response to therapies to refine patient selection and maximise PARPi benefit.

## Data Availability

Not applicable.
